# Continuous veno-venous hemofiltration yields better renal outcomes than intermittent hemodialysis among traumatic intracranial hemorrhage patients with acute kidney injury: A nationwide population-based retrospective study in Taiwan

**DOI:** 10.1371/journal.pone.0203088

**Published:** 2018-09-20

**Authors:** Min-Feng Tseng, Chu-Lin Chou, Chi-Hsiang Chung, Wu-Chien Chien, Ying-Kai Chen, Hsiu-Chien Yang, Chen-Yi Liao, Kuang-Yu Wei, Chia-Chao Wu

**Affiliations:** 1 Department of Internal Medicine, Zuoying Branch of Kaohsiung Armed Forces General Hospital, Kaohsiung, Taiwan; 2 Division of Nephrology, Department of Internal Medicine, Tri-Service General Hospital, National Defense Medical Center, Taipei, Taiwan; 3 Division of Nephrology, Department of Internal Medicine, Shuang Ho Hospital, Taipei Medical University, New Taipei City, Taiwan; 4 Division of Nephrology, Department of Internal Medicine, School of Medicine, College of Medicine, Taipei Medical University, Taipei, Taiwan; 5 School of Public Health, National Defense Medical Center, Taipei, Taiwan; 6 Taiwanese Injury Prevention and Safety Promotion Association, Taipei, Taiwan; 7 Division of Nephrology, Department of Internal Medicine, Kaohsiung Armed Forces General Hospital, Kaohsiung, Taiwan; National Yang-Ming University, TAIWAN

## Abstract

**Object:**

Traumatic intracranial hemorrhage (TICH) patients with acute kidney injury (AKI) were reported to have a high mortality rate. Renal replacement therapy (RRT) is indicated for patients with a severe kidney injury. This study aimed to compare the effects of different RRT modalities regarding chronic dialysis rate among adult TICH patients with AKI.

**Methods:**

A retrospective search of computerized hospital records from 2000 to 2010 for patients with a discharge diagnosis of TICH was conducted to identify the index cases. We collected the data of TICH patients with increased intracranial pressure combined with severe AKI who received intermittent hemodialysis (IHD) or continuous veno-venous hemofiltration (CVVH) as RRT. The outcome was dialysis dependence between 2000 and 2010.

**Results:**

From a total of 310 patients who were enrolled in the study, 134 (43%) received CVVH and 176 (57%) received IHD. The risk of dialysis dependency was significantly lower in the CVVH group than in the IHD group (adjusted hazard ratio: 0.368, 95% CI, 0.158–0.858, P = 0.034). Diabetes mellitus and coronary artery disease were risk factors for dialysis dependency. CVVH compared with IHD modality was associated with lower dialysis dependency rate in TICH patients combined with AKI and diabetes mellitus and those with an injury severity score (ISS) ≥16.

**Conclusion:**

CVVH may yield better renal outcomes than IHD among TICH patients with AKI, especially those with diabetes mellitus and an ISS ≥16. The beneficial impact of CVVH on TICH patients needs to be clarified in a large cohort study in future.

## Introduction

There is little information available on the outcome comparison of acute kidney injury (AKI) in patients with traumatic intracranial hemorrhage (TICH). In severe TICH, the hematoma lesion can compress the brain and increase the intracranial pressure, causing conscious disturbance and potentially fatal herniation syndromes. TICH remains a leading cause of death and persistent neurocognitive impairment. Recent studies focusing on non-neurologic organ dysfunction in patients with severe traumatic brain injury have demonstrated that nearly 0.5% of these patients develop AKI, which is independently associated with worse outcomes.[1–3]

TICH patients suffer a range of complications, including thromboembolism, respiratory infection, and neurologic dysfunction, due to immobilization.[4]

These are believed to commonly contribute to renal complications during hospitalization. Patients with traumatic intracerebral hemorrhage are absolutely susceptible to acute renal injury due to trauma-induced volume depletion, which decreases renal perfusion. Other possible AKI findings suggest the contribution of computed tomography angiography to the development of nephropathy or occurrence of transtentorial herniation or nephrotoxic antibiotics after TICH. The incidence of contrast-induced nephropathy has been reported to be 2–3% in patients with cerebrovascular emergencies.[5–7]

AKI is an increasingly and potentially catastrophic complication among TICH patients, and survivors are at an increased risk for chronic kidney disease (CKD), end-stage renal disease (ESRD) requiring long-term renal replacement therapy, or later death.[8–12] It is worth noting that mortality rates of critically ill patients with AKI still have remained high around 50%.[13]

Renal replacement therapy (RRT) in patients with AKI corrected acid-base and electrolyte imbalance, ameliorated volume overload, and removed uremic toxin. Although RRT can forestall or reverse the life-threatening complications of AKI, it does not hasten and can potentially delay the recovery of kidney function in patients with AKI and can be associated with potentially life-threatening complications. Intermittent hemodialysis (IHD) was originally conceived as a treatment for acute renal failure, and continuous renal replacement therapy (CRRT) was introduced as an alternative when IHD was contraindicated. With a greater hemodynamic tolerance than IHD, CRRT appears to preserve organ function and survival.

Many studies have explored to adjudge the optimal modality for RRT treatment of critically ill patients with AKI. The results of observational studies, randomized clinical trials, and meta-analyses comparing these procedures have failed to demonstrate the superiority of either CRRT or IHD regarding mortality.[14–17]

However, to the best of our knowledge, there is no study focusing on the outcome among TICH patients receiving CRRT versus IHD. In this analysis, we used the National Health Insurance Research Database (NHIRD) and designed a retrospective cohort study to compare the outcomes between CVVH and the conventional IHD treatment in TICH patients with AKI.

## Materials and methods

### Data sources

This study was approved by the Institutional Review Board of the Tri-Service General Hospital (No. 2-105-05-082). The study enrolled all patients diagnosed with TICH who received glycerol or mannitol and required emergent hemodialysis in Taiwan to minimize selection bias. Glycerol or mannitol has been applied in raised intracranial pressure according to Taiwan National Health Insurance (NHI) Referral Guidelines. In this study, we used data from the NHIRD in Taiwan to investigate the mortality rate, length of hospital stay, medical cost, and renal outcome within a 10-year period, specifically data from 2000 to 2010.[18]

The NHI program has provided compulsory universal health insurance for all citizens (23.74 million residents), and it covers more than 99% of the population of Taiwan. Hence, claims data obtained from the NHIRD are ideal for longitudinal cohort studies.[19–22] The diagnosis codes are based on the ninth revision of the International Classification of Diseases. For privacy protection, the individuals’ identities are encrypted within the NHI database. The initial severity of TICH as determined by the injury severity score (ISS) was recorded at the time of ICU admission during the first 24 h.

### Study cohort and patient selection

This study has a retrospective cohort design. All patients were older than 18 years and were diagnosed with TICH between 2000 and 2010 (subarachnoid, subdural, and extradural hemorrhage ICD-9-CM codes: 800.2, 800.7, 801.2, 801.7, 803.2, 803.7, 804.2, 804.7; intracranial hemorrhage ICD-9-CM codes: 800.3, 800.8, 801.3, 801.8, 803.3, 803.8, 804.3, 804.8). The pathophysiology and management of TICH in children and patients younger than 18 years old are quite different from those in adults. The differences are attributable to age-related structural change, mechanism of injuries based on physical ability of the child, and the difficulty in neurological evaluation of pediatric populations. The scalp is highly vascularized and a potential cause of lethal blood loss. Even a small loss of blood volume can lead to hemorrhagic shock in a newborn, infant, and toddler, which may occur without apparent external bleeding. Hence, we only enrolled patients older than 18 years old. The main exclusion criteria at the time of enrollment included TICH diagnosed before 2000, hemodialysis before TICH, underlying disease including CKD, malignancy, coagulation defects, purpura and other hemorrhagic conditions, mortality/mechanical ventilation/ischemic heart disease before tracking, patients with CKD receiving chronic RRT, status post kidney transplant, and patients receiving different modalities of RRT during the same hospital admission. Furthermore, patients with mechanical ventilation may increase incidence of sepsis which frequently induce acute kidney injury. On the other hand, the existence of ischemic heart diseases may also appear to increase the risk of acute kidney injury which may influence our result. Hence, we excluded these populations to minimize confounding factors. If a patient had several episodes in the intensive care unit of AKI-RRT during the same hospital admission, only the first episode was considered.

During the study period, the medical history (hypertension, hyperlipidemia, stroke, diabetes mellitus, coronary artery disease, heart failure, peripheral vascular disease), antiplatelet medication (clopidogrel, ticagrelor, prasugrel, dipyridamole, aspirin, ticlopidine, eptifibatide), anticoagulant medication (heparin, warfarin, rivaroxaban, dabigatran, apixaban, edoxaban, enoxaparin, fondaparinux), presence or absence of head injury and the type of surgery, Charlson comorbidity index[23], length of days, medical cost, and level of care were recorded at admission.

RRT included IHD (ICD-9-CM code: 58001) and CRRT including continuous veno-venous hemofiltration (CVVH) (ICD-9-CM code: 58014C). The RRT procedures were determined by consensus between the attending intensivists and nephrologists based on the clinical status of the patient (fluid balance, respiratory status, acid-base balance).

### Outcome measures

The outcome was the numbers of AKI cases treated using different RRT modalities with hemodialysis dependency. The initiation of dialysis dependency was defined as the date of starting dialysis for at least three consecutive months or the approval date of the catastrophic illness certificate for end-stage renal disease (ESRD), whichever came first, to assure actual need and receipt of dialysis. Modes of dialysis included hemodialysis (ICD-9-CM codes: 58027C, 58029C). The condition of individuals with CKD, who require long-term hemodialysis, is referred to as dialysis dependency or ESRD. All the study participants were followed up from the index date to the onset of dialysis dependency (ICD-9-CM code: 58027C or 58029C) or withdrawal from the NHI program, death, or the end of 2010.

### Statistical analysis

All data were analyzed using the SAS statistical software (SAS System for Windows, version 9.1.3; SAS Institute, Cary, NC, USA). χ2 and t-test were used to evaluate the differences in age and comorbidities between the IHD and CVVH group. The Fisher exact test for categorical variables was used to statistically examine the differences between the two cohorts. The Cox proportional regression hazard model was used to compare the incidence rate of dialysis dependency between the IHD and CVVH group after the modification of comorbidities. The Kaplan–Meier method and log-rank test were used to estimate the outcome of the two groups. A two-tailed p-value level lesser than 0.05 was considered significant.

## Results

### Characteristics of the study population

**Fig 1:**
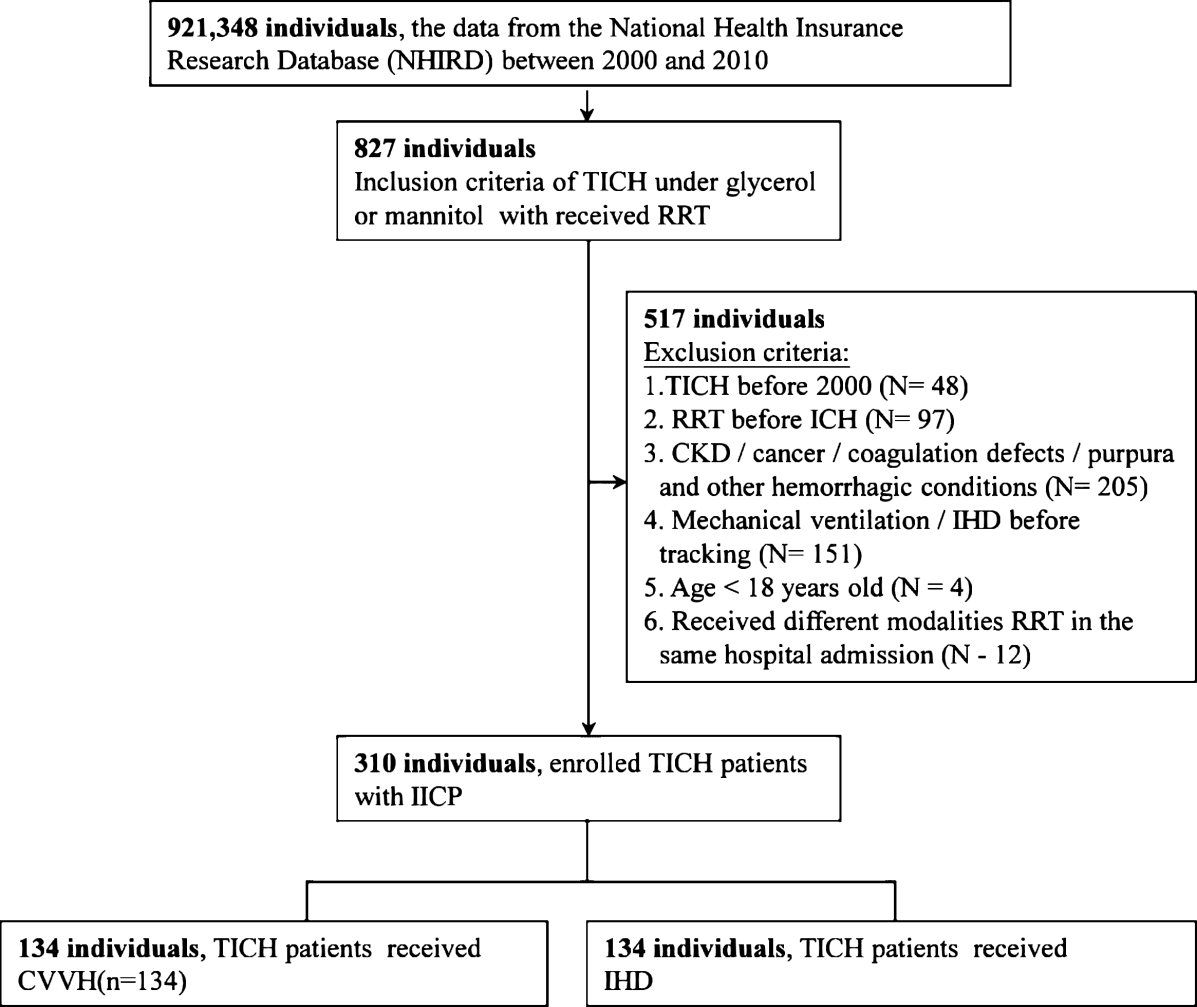
Cohort assembly in this study. CKD, Chronic kidney disease; RRT, Renal replacement therapy; CVVH, Continuous venovenous hemofiltration; IHD, Intermittent hemodialysis; IICP, Increased intracranial pressure.

During the 10-year study period, a total of 827 TICH patients with AKI requiring RRT were enrolled. A total 310 patients were included in the final analysis (Fig 1). Table 1 shows the baseline characteristics of the enrolled patients receiving IHD or CVVH. Among the eligible patients, there were no baseline differences in sex, age, ISS, comorbidities (diabetes mellitus, hypertension, hyperlipidemia, stroke, coronary artery disease, heart failure, peripheral vascular disease), Charlson comorbidity index, use of baseline medications including antiplatelet and anticoagulant drugs, level of care, head surgery, length of days, and medical cost (NT$).

**Table 1:** Characteristics of study in the baseline.

*Hemodialysis methods*	*Total*		*IHD*		*CVVH*		*P*
*Variables*	*n*	*%*	*n*	*%*	*n*	*%*	
*Total*	*310*		*176*	*56*.*77*	*134*	*43*.*23*	
*Gender*							*0*.*062*
*Male*	*238*	*76*.*77*	*142*	*80*.*68*	*96*	*71*.*64*	
*Female*	*72*	*23*.*23*	*34*	*19*.*32*	*38*	*28*.*36*	
*Age (years)*	*43*.*46±19*.*30*		*42*.*95±19*.*41*		*44*.*11±19*.*21*		*0*.*600*
*ISS≧16*							*0*.*133*
*Without*	*218*	*70*.*32*	*130*	*73*.*86*	*88*	*65*.*67*	
*With*	*92*	*29*.*68*	*46*	*26*.*14*	*46*	*34*.*33*	
*DM*							*0*.*319*
*Without*	*300*	*96*.*46*	*171*	*97*.*16*	*129*	*95*.*56*	
*With*	*11*	*3*.*54*	*5*	*2*.*84*	*6*	*4*.*44*	
*HT*							*0*.*385*
*Without*	*196*	*93*.*33*	*167*	*94*.*89*	*29*	*85*.*29*	
*With*	*14*	*6*.*67*	*9*	*5*.*11*	*5*	*14*.*71*	
*Hyperlipidemia*							*-*
*Without*	*310*	*100*.*00*	*176*	*100*.*00*	*134*	*100*.*00*	
*With*	*0*	*0*.*00*	*0*	*0*.*00*	*0*	*0*.*00*	
*Stroke*							*0*.*399*
*Without*	*307*	*99*.*03*	*175*	*99*.*43*	*132*	*98*.*51*	
*With*	*3*	*0*.*97*	*1*	*0*.*57*	*2*	*1*.*49*	
*CAD*							*0*.*080*
*Without*	*307*	*99*.*03*	*176*	*100*.*00*	*131*	*97*.*76*	
*With*	*3*	*0*.*97*	*0*	*0*.*00*	*3*	*2*.*24*	
*HF*							*0*.*568*
*Without*	*309*	*99*.*68*	*175*	*99*.*43*	*134*	*100*.*00*	
*With*	*1*	*0*.*32*	*1*	*0*.*57*	*0*	*0*.*00*	
*PVD*							*-*
*Without*	*310*	*100*.*00*	*176*	*100*.*00*	*134*	*100*.*00*	
*With*	*0*	*0*.*00*	*0*	*0*.*00*	*0*	*0*.*00*	
*CCI*	*0*.*06±0*.*27*		*0*.*07±0*.*30*		*0*.*04±0*.*24*		*0*.*455*
*Anti-platelet drug*							*0*.*786*
*Without*	*250*	*80*.*65*	*141*	*80*.*11*	*109*	*81*.*34*	
*With*	*60*	*19*.*35*	*35*	*19*.*89*	*25*	*18*.*66*	
*Anti-coagulant drug*							*0*.*389*
*Without*	*252*	*81*.*29*	*146*	*82*.*95*	*106*	*79*.*10*	
*With*	*58*	*18*.*71*	*30*	*17*.*05*	*28*	*20*.*90*	
*Level of care*							*0*.*550*
*Hospital center*	*130*	*41*.*94*	*78*	*44*.*32*	*52*	*38*.*81*	
*Regional hospital*	*154*	*49*.*68*	*85*	*48*.*30*	*69*	*51*.*49*	
*Local hospital*	*26*	*8*.*39*	*13*	*7*.*39*	*13*	*9*.*70*	
*Head surgery*							*0*.*151*
*Without*	*155*	*50*.*00*	*93*	*52*.*84*	*62*	*46*.*27*	
*With*	*155*	*50*.*00*	*83*	*47*.*16*	*72*	*53*.*73*	
*Length of hospital stay*	*20*.*47±15*.*50*		*20*.*33±16*.*48*		*20*.*65±14*.*15*		*0*.*858*
*Medical cost (NT$)*	*215*,*554*.*41±164*,*883*.*40*		*208*,*974*.*24±107*,*130*.*29*		*224*,*197*.*01±157*,*941*.*51*		*0*.*422*

P-value (category variable: Chi-square/Fisher exact test; continue variable: t-test)

CRRT = Continuous venovenous hemofiltration; IHD = Intermittent hemodialysis

ISS = Injury severity score

DM = Diabetes mellitus; HT = Hypertension; CAD = Coronary artery disease; HF = Heart failure; PVD

A total of 134 patients (43%) received CVVH as the initial RRT. Follow-up data at ten years were available for 296 patients (95%); a total of 14 deaths had been observed by year 10 (9 in the IHD group and 5 in the CVVH group).

### Long-term hemodialysis rate in TICH patients using different RRT

**Fig 2:**
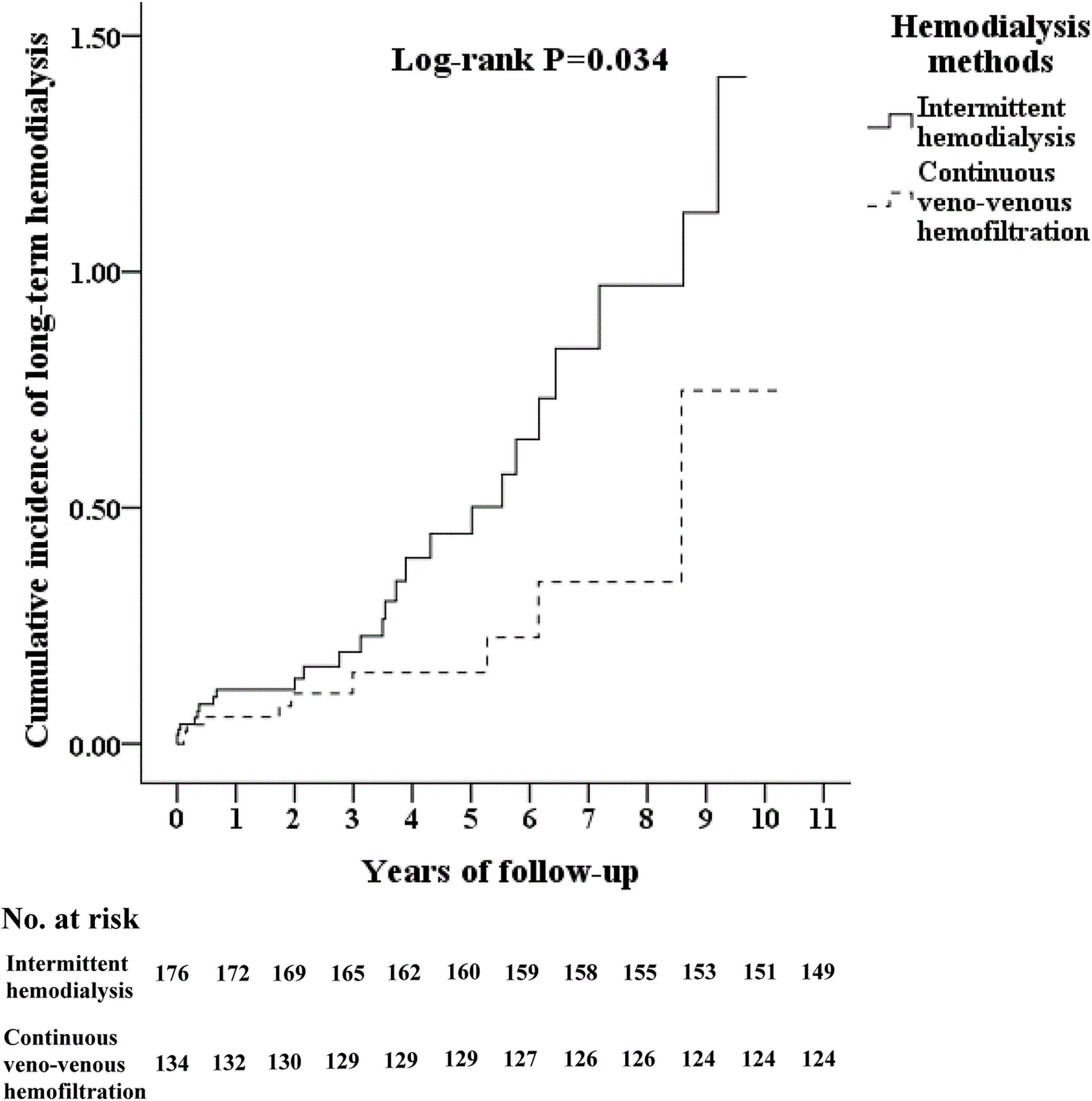
Kaplan-Meier curves for the cumulative incidence of dialysis dependency stratified by hemodialysis methods with log-rank test model in traumatic intracranial hemorrhage patients with acute kidney injury treated with intermittent hemodialysis or continuous veno-venous hemofiltration of renal-replacement therapy. CVVH, Continuous venovenous hemofiltration; IHD, Intermittent hemodialysis.

The Kaplan–Meier method for the cumulative incidence of dialysis dependency demonstrated significant differences between the IHD group and the CVVH group (P = 0.034). The dialysis dependency rate of the IHD group was higher than that of the CVVH group. A total of 37 patients required long-term dialysis (27 in the IHD group and 10 in the CVVH group) (Fig 2).

### Risk factors of long-term hemodialysis in TICH patients combined with severe AKI

Using the Cox model with competing risks, diabetes mellitus (95% CI = 1.387–23.086, adjusted hazard ratio (HR): 5.658, P = 0.016) and coronary artery disease (95% CI = 1.204–36.851, adjusted HR: 6.662, P = 0.030) were shown to have associations with increased risk for chronic dialysis (Table 2).

**Table 2:** Hazard ratio of chronic dialysis in association with baseline characteristics among traumatic intracranial hemorrhage patients with acute kidney injury in Cox model with competing risks.

Variables	Adjusted HR	95% CI	95% CI	P
Hemodialysis methods				
IHD	Reference			
CVVH	0.368	0.158	0.858	0.021*
Gender				
Male	1.312	0.439	3.922	0.627
Female	Reference			
Age (years)	1.016	0.996	1.037	0.123
ISS≧16				
Without	Reference			
With	1.629	0.657	4.402	0.192
DM				
Without	Reference			
With	5.658	1.387	23.086	0.016*
HT				
Without	Reference			
With	0.113	0.015	1.182	0.070
Stroke				
Without	Reference			
With	1.120	0.206	6.084	0.896
CAD				
Without	Reference			
With	6.662	1.204	36.851	0.03*
CCI	0.979	0.864	1.218	0.772
Level of care				
Hospital center	1.022	0.381	2.742	0.966
Regional hospital	0.410	0.145	1.161	0.093
Local hospital	Reference			
Head surgery				
Without	Reference			
With	2.266	0.617	8.312	0.218
Length of hospital stay	1.006	0.957	1.038	0.858

HR = hazard ratio, CI = confidence interval, Adjusted HR: Adjusted variables listed in the table

CVVH = continuous veno-venous hemofiltration; IHD = intermittnet hemodialysis; ISS = injury severity score; DM = diabetes mellitus; HT = hypertension; CAD = coronary artery disease; HF = heart failure; PVD = peripheral vascular disease; CCI = charlson comorbility index

*denotes P < .05 and was considered statistically significant

### Cumulative risk of long-term hemodialysis in TICH patients combined with diabetes mellitus and severe AKI using different RRT

**Fig 3:**
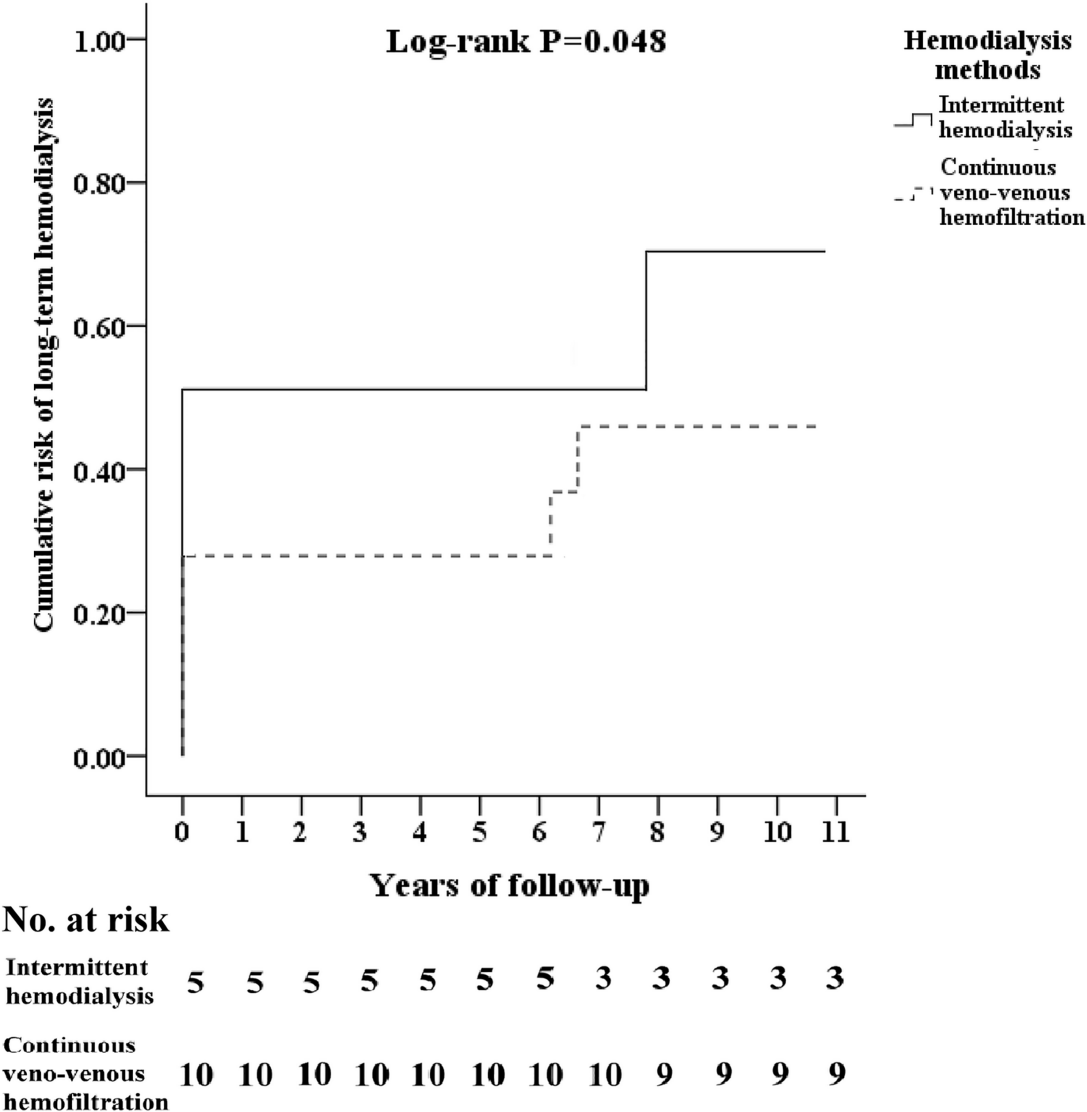
Kaplan-Meier curves for the cumulative incidence of dialysis dependency stratified by hemodialysis methods with log-rank test model in traumatic intracranial hemorrhage patients combined with acute kidney injury and diabetes mellitus treated with intermittent hemodialysis or continuous veno-venous hemofiltration of renal-replacement therapy. CVVH, Continuous venovenous hemofiltration; IHD, Intermittent hemodialysis.

Fig 3 shows the cumulative risk of long-term hemodialysis in TICH patients combined with diabetes mellitus and severe AKI using different RRT. These results show that IHD compared with CVVH had lower long-term hemodialysis rate in TICH patients combined with diabetes mellitus and severe AKI. The following time and years to long-term hemodialysis were showed in S1 Table. We also tracked TICH patients combined with coronary artery disease and severe AKI using different RRT, long-term dialysis events in CVVH and IHD group were 2 and 0, respectively.

### Subanalysis outcome (IHD versus CVVH)

Furthermore, when we focused on factors of chronic dialysis stratified by variables using Cox regression, CVVH results showed beneficial effect in terms of ISS ≧16 (95% CI = 0.155–0.839, adjusted HR: 0.358, P = 0.020) and tracking period ≧1 year (95% CI = 0.101–0.498, adjusted HR: 0.231, P = 0.001) compared with IHD results (Table 3).

**Table 3:** Factors of dialysis dependancy stratified by variables listed in the table by using Cox regression.

	CVVH					IHD	Adjusted HR (CVVH versus IHD)	P
Stratified	Event	PYs	Rate (per 10^5^ PYs)	Event	PYs	Rate (per 10^5^ PYs)		
Total	10	815.23	1,226.65	27	963.11	2,803.42	0.368	0.021*
Gender								
Male	6	593.66	1,010.68	23	831.41	2,766.38	0.307	0.452
Female	4	221.57	1,805.30	4	131.70	3,037.21	0.500	0.334
ISS≧16								
Without	6	615.15	975.37	19	792.98	2,396.03	0.315	0.143
With	4	200.08	1,999.20	8	170.13	4,702.29	0.358	0.02*
Head surgery								
Without	8	754.32	1,060.56	25	889.15	2,811.67	0.317	0.206
With	2	60.91	3,283.53	2	73.96	2,704.16	0.921	0.595
Tracking time								
≦30 days	0	1.02	0.00	4	8.84	45,248.87	0.000	0.842
>31 days, <1 year	4	41.03	9,748.96	5	351.28	1,423.37	5.760	0.297
≧1 year	6	733.19	818.34	18	602.99	2,985.12	0.231	0.001*

PY = Person-years; Adjusted HR = Adjusted Hazard ratio: Adjusted for gender, age, comorbities, medicine, season, urbanization, level of care, and insured premium; CI = confidence interval; CVVH = Continuous veno-venous hemofiltration; IHD = Intermittent hemodialysis; ISS = Injury severity score

## Discussion

The present study was the first to examine the outcome comparison of CVVH and IHD in TICH patients with AKI complications. After multivariate adjustment and subgroup analysis, the major findings of our study are outlined as follows: (1) CVVH compared with IHD as the initial RRT modality was associated with decreased risk for dialysis dependency. (2) Among TICH patients with AKI, diabetes mellitus and coronary artery disease were risk factors for dialysis dependency. (3) Compared with IHD, the rates of dialysis dependency was significantly lower in TICH patients with AKI and diabetes mellitus receiving CVVH. (4) CVVH in patients with more severe TICH (injury severity ≥16) appeared to have protective effects against dialysis dependency in CKD patients.

Hemofiltration is most commonly used in an intensive care unit setting; this is possibly related to the theoretical advantage of increased clearance of higher molecular weight molecules and inflammatory cytokines, especially in septic patients.[24] In our study, CVVH as the initial RRT modality was associated with decreased risk of chronic dialysis. These findings demonstrated that renal injury is closely associated with hemodynamics, and the use of CVVH for accurate quantitative volume management can, therefore, help improve renal perfusion.[25] Tang et al. demonstrated that CVVH improved mitochondrial function and reduced the renal tubular epithelial apoptosis. [26]

Furthermore, another recent study on acute brain injury requiring RRT also demonstrated that IHD might exacerbate the injury by compromising cerebral perfusion pressure, either after a reduction in cerebral perfusion or increased cerebral edema. A retrospective observational cohort study of patients with acute brain injury showed CRRT might have beneficial effects in patients with refractory intracranial hypertension associated with high mortality rate and poor neurological outcome.[27] In contrast, IHD can induce hypovolemia and, consequently, may worsen renal ischemic injury, delay recovery of renal functions especially in patients with high ISS, and result in a need for prolonged dialysis.[28] In our study, we also found that the use of CVVH in patients with more severe TICH (ISS ≧16) resulted in lower dialysis dependency. These phenomena are due to the beneficial effect of CVVH that can be attributed to the allowance of better fluid clearance and regulation of volume status with a resultant reduction in the intracerebral pressure in TICH patients, which may reduce the need for prolonged administration of mannitol or glycerol.

In this retrospective analysis of the NHIRD, we found that diabetes mellitus and coronary artery disease were risk factors for dialysis dependency. Previous studies also demonstrated that the risk factors among non-recovery AKI patients, including comorbidities such as hypertension, diabetes mellitus, and cardiac disease, are the most frequently reported problems.[29–30] Chronic kidney disease may potentially result from end-organ damage such as coronary artery disease-induced heart failure related renal hypoperfusion or diabetes-related generalized and intrarenal atherosclerosis. A retrospective analysis also revealed diabetes as an independent risk factor for developing AKI.[31] Further, a retrospective study showed that AKI patients are at risk for the development of CKD 1 year following hospitalization.[32] We found that CVVH compared with IHD modality in TICH patients combined with AKI and diabetes mellitus was associated with lower dialysis dependency rate. Vallon speculates that diabetic tubular growth and upregulation of TGF-β, senescence, and inflammation in diabetes mellitus increased the susceptibility to AKI, which further promotes hypoxia and apoptosis. [33] Previous clinical and experimental evaluates the ability of CVVH to eliminate small and medium-sized inflammatory mediators such as cytokines. [34,35] De Vriese et al. demonstrated that cytokine removal via hemofiltrate is associated with a significant decrease of cytokine plasma levels. Also, there was a close relationship between both the filtration rate and frequency of filter changes with the efficiency of CVVH in removing cytokines. [36] Other studies reported the benefits of increasing the filtration rate regarding survival improvement and accelerated recovery from AKI. [37] According to these results, Claudio Ronco et al. recommend a filtration rate of at least 35 mL/kg BW/h. [38] Finally, It has been hypothesized that CVVH may prevent renal microcirculation deterioration in AKI patient complicated with DM and TICH.

There are limitations to our findings. First, because there is no link between the NHIRD and Glasgow Coma Scale level, we did not have access to information regarding consciousness. Second, the NHIRD provided no detailed information regarding head surgery, such as hematoma area and size, blood loss during surgery, or anesthesia time. This precluded us from performing any analysis involving these variables. Third, the NHIRD provided no further information about IHD and CVVH, such as dialysis flow rate, dialysis time or duration, and ultrafiltration amount. This pattern may have influenced the results obtained. Fourth, the facilities in different levels of hospitals may be different. However, the renal replacement procedures were all performed by nephrologist who had received well training and qualified in Taiwan. They all have validated experience in the management of AKI and techniques of RRT. Furthermore, there were no significant different distributions of different levels of hospital between the two groups. Hence, it is less likely that different levels of hospitals will significantly interfere with our result. Fifth, CVVH is more suitable for patient with unstable hemodynamics than IHD. It is possible that selection bias may exist between the two groups. However, our data are identified and obtained according the ICD 9 code from National Health Insurance Research Database (NHIRD) which includes almost all cases of traumatic intracranial hemorrhage and CVVH / TICH use in the random source population. The loss-to-follow-up rate is low, due to its compulsive characteristics, high coverage of the entire Taiwan population and management by the government. Since there has been no significant change in ICD- 9 codes and indications for acute dialysis, it is unlikely that the data quality and renal outcome would have changed significantly. Furthermore, we had adjusted variables including age, sex, injury severity score and comorbidities such as diabetes mellitus, hypertension, coronary artery disease, stroke, heart failure, and peripheral vascular disease in our study. Hence, it is less likely that selection bias will significantly interfere with our result.

## Conclusion

Our study is large-scale nationwide cohort study investigating the outcomes among TICH patients with AKI. We concluded that CVVH might yield better renal outcomes than IHD in TICH patients with AKI, especially those with diabetes mellitus and an ISS ≥16. The beneficial impact of CVVH on TICH patients need to be clarified in a large cohort study in future.

## Supporting information

S1 Table (DOCX)
