# Supplementary material for: Continuous veno-venous hemofiltration yields better renal outcomes than intermittent hemodialysis among traumatic intracranial hemorrhage patients with acute kidney injury: A nationwide population-based retrospective study in Taiwan
Source: PLoS One. 2018 Sep 20;13(9):e0203088. doi: 10.1371/journal.pone.0203088 (PMC6157819; doi:10.1371/journal.pone.0203088)
Supplement: S1 Table — (DOCX) [file pone.0203088.s001.docx]

**S1 Table. Years of follow-up and years to long term hemodialysis among TICH patients with DM and AKI**

|  | **IHD** | |  | | **CVVH** |  |
| --- | --- | --- | --- | --- | --- | --- |
| Years of follow-up | 5.99±9.15 |  |  |  | 5.42±9.28 |  |
| Years to long term hemodialysis | 6.33 |  |  |  | 7.83 |  |

TICH = Traumatic intracranial hemorrhage

DM = Diabetes mellitus

AKI = Acute kidney injury

IHD = Intermittent hemodialysis

CVVH = Continuous veno-venous hemofiltration
